# Topping-off surgery vs posterior lumbar interbody fusion for degenerative lumbar disease: a finite element analysis

**DOI:** 10.1186/s13018-019-1503-4

**Published:** 2019-12-30

**Authors:** Yunpeng Fan, Shaobo Zhou, Tao Xie, Zefeng Yu, Xiao Han, Liulong Zhu

**Affiliations:** 1 Department of Orthopedic Surgery, The Affiliated Hangzhou Hospital of Nanjing Medical University, Hangzhou, 310006 China; 2 The Affiliated Hangzhou First People’s Hospital, Zhejiang University School of Medicine, Hangzhou, 310006 China

**Keywords:** Coflex, PLIF, Adjacent segment disease, ASD, Finite element analysis

## Abstract

**Background:**

Adjacent segment disease (ASD) is a common complication after posterior lumbar interbody fusion (PLIF). Recently, a topping-off surgery (non-fusion with Coflex) has been developed to reduce the risk of ASD, yet whether and how the topping-off surgery can relieve ASD remains unclear. The purpose of this study was to explore the biomechanical effect of PLIF and Coflex on the adjacent segments via finite element (FE) analysis and discuss the efficacy of Coflex in preventing ASD.

**Methods:**

A FE model of L3–L5 segments was generated based on the CT of a healthy volunteer via three commercially available software. Coflex and PLIF devices were modeled and implanted together with the segment model in the FE software. In the FE model, a pre-compressive load of 500 N, equal to two-thirds of the human body mass, was applied on the top surface of the L3. In addition, four types of moments (anteflexion, rear protraction, bending, and axial rotation) set as 10 Nm were successively applied to the FE model combined with this pre-compressive load. Then, the range of motion (ROM), the torsional rigidity, and the maximum von Mises equivalent stress on the L3–L4 intervertebral disc and the implant were analyzed.

**Results:**

Both Coflex and PLIF reduced ROM. However, no significant difference was found in the maximum von Mises equivalent stress of adjacent segment disc between the two devices. Interestingly enough, both systems increased the torsional rigidity at the adjacent lumbar segment, and PLIF had a more significant increase. The Coflex implant had a larger maximum von Mises equivalent stress.

**Conclusions:**

Both Coflex and PLIF reduced ROM at L3–L4, and thus improved the lumbar stability. Under the same load, both devices had almost the same maximum von Mises equivalent stress as the normal model on the adjacent intervertebral disc. But it is worthy to notice the torsional rigidity of PLIF was higher than that of Coflex, indicating that the lumbar treated with PLIF undertook a larger load to reach ROM of Coflex. Therefore, we presumed that ADS was related to a higher torsional rigidity.

## Background

Lumbar spinal stenosis (LSS), a common senile disease, has a 9.3% incidence in the elderly [1]. LSS is a major cause of surgery in people over 65 [2]. Clinical observations show the onset age of LSS is becoming younger on account of the sedentary lifestyle. LSS is clinically manifested by numbness and radiative pain in the buttocks and lower limbs [3]. Some patients may have lower back pain. These symptoms exert negative impacts on the patient’s physical functions. Some bedridden LSS patients even develop pneumonia and deep vein thrombosis (DVT).

Posterior lumbar interbody fusion (PLIF), as the “gold standard” in surgical practice, can significantly relieve the symptoms of nerve root compression. Although the primary segment disc compression had a low recurrence rate after PLIF, during the long-term follow-up, many patients develop LSS at an adjacent segment and recurrent relative nerve root compression. Therefore, PLIF surgery may increase the incidence of adjacent segment disease (ASD) [3, 4]. Currently, a new “topping-off” technique using Coflex, a U-shaped elastic non-fusion interspinous instrument placed between two adjacent spinous processes, is often applied in clinical practice. Some literature has demonstrated Coflex implantation can reduce the risk of ASD [3, 4].

Colfex has been proven safe and effective for LSS. However, the effect of the Coflex on the adjacent segment is still not clear. Lee [4] believed ASD after PLIF was caused by the compensatory increase in the mobility of the adjacent lumbar segment and the added pressure on the intervertebral disc and intervertebral joints. We presumed that torsional rigidity might be related to ASD, but the torsional rigidity was rarely addressed in the previous studies. To figure out the specific mechanism of how Coflex reduced the rate of ASD and the relationship between torsional rigidity and ASD, we conducted this study to explore the biomechanical effect between PLIF and Coflex on the adjacent segments via finite element analysis and discuss the efficacy of Coflex in preventing ASD.

## Methods

### Establishment of basic model

**Fig. 1:**
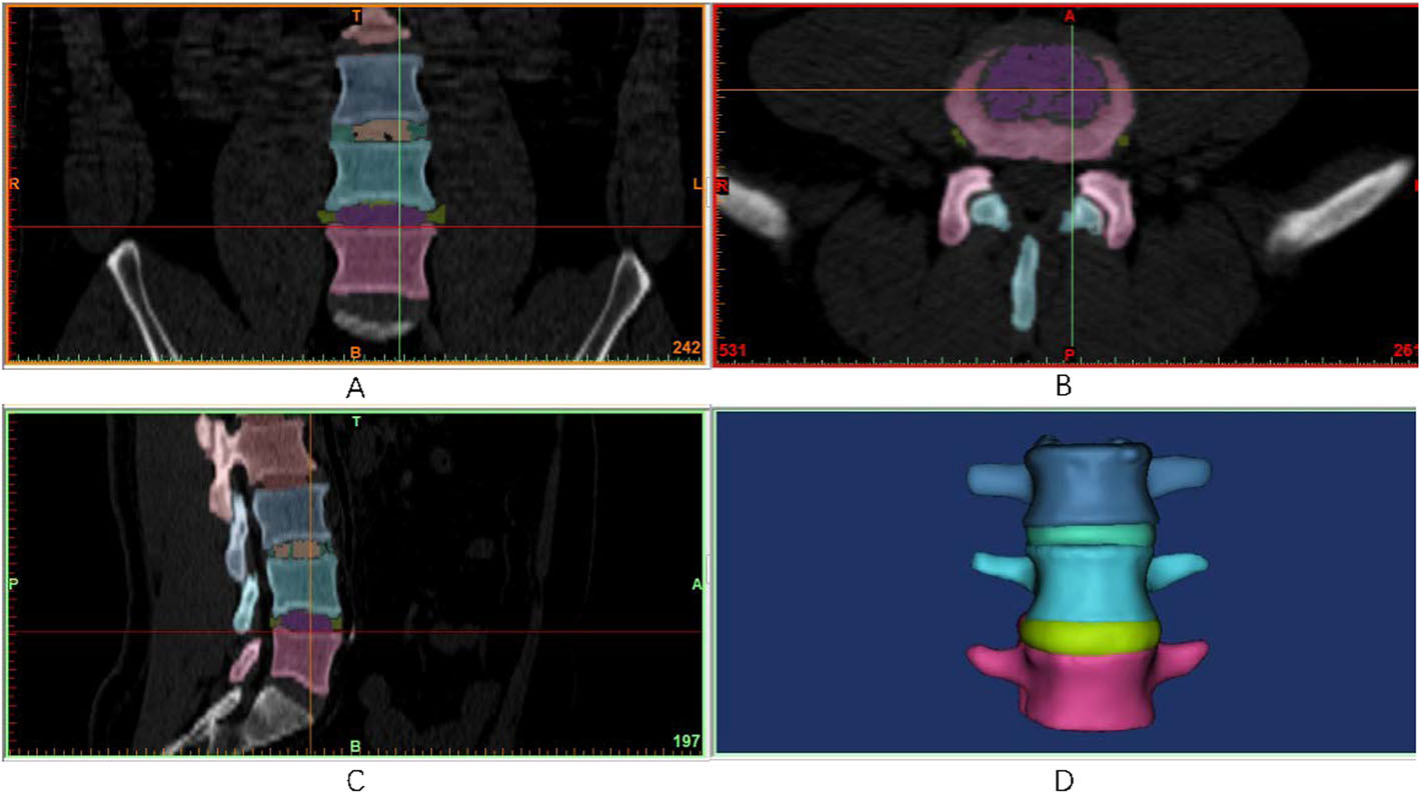
**a**, **b**, **c** The mask and 3D model drawing process of intervertebral disc, nucleus and vertebral body mask. **d** The mask and 3D model drawing process of 3D solid model

The geometrical specifications of the spine were obtained from 64 spiral CT images of a 28-year-old male without spine injury or radiographic evidence of degeneration. He received a SOMATOM SENSATION 64 spiral CT (Siemens, Munich, Germany) scan for health examination at our hospital. The CT images were used with his consent. The CT scans of the L3–L5 lumbar spine with Coflex or PLIF were obtained at 1-mm intervals. The threshold segmentation was used in MIMICS19 based on the CT data to obtain the masks of the intervertebral disc, nucleus pulposus, vertebral body, and the implant. Using the masks, the 3-D solid model was constructed (Fig. 1). The contour of the model was refined and the CT image distortion was decreased as much as possible. Then, the STL file was output with the quality of the mesh maintained. In GEOMAGIC12, the STL vertebral model was de-noised and smoothed with all the details maintained. All vertebral models were offset by 1 mm to make a smaller vertebral cancellous bone model. The STP files of all models were output and imported into UG software, and then, the final models were obtained by Boolean subtraction [5–9].

**Fig. 2:**
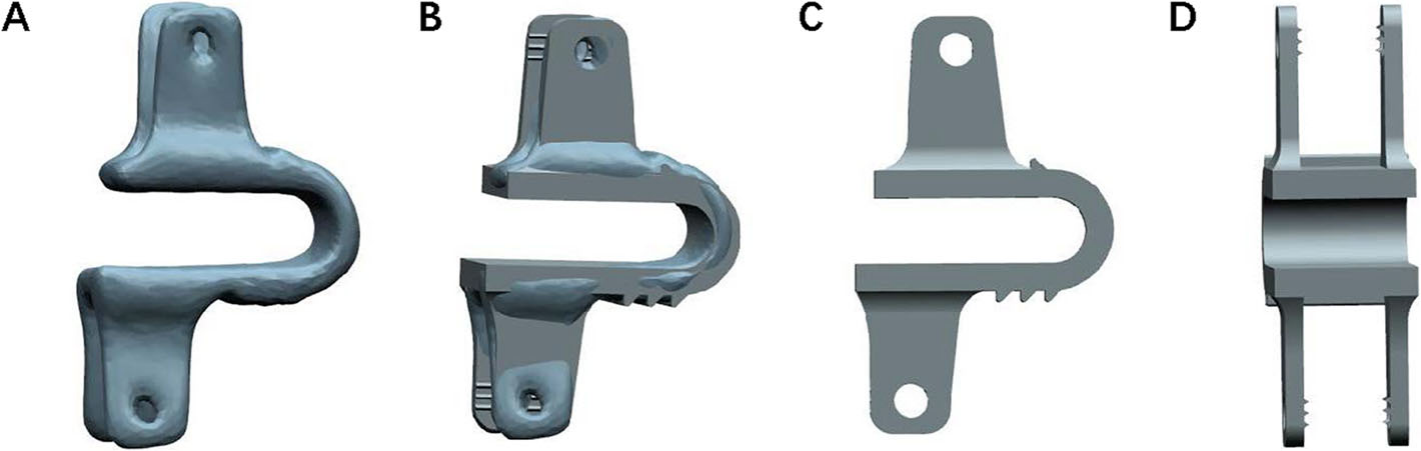
**a**, **b** The model of the Coflex solid model. **c**, **d** The model of the Coflex formal model

Threshold segmentation was used in MIMICS19 based on postoperative CT to obtain the Coflex implant masks. The preliminary solid model was established by 3-D model construction using the masks. The output STL file was imported into the UG software, and the Coflex model was designed based on the preliminary model using the UG software (Fig. 2).

**Fig. 3:**
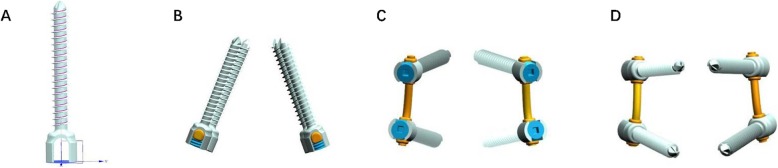
**a**, **b** The K-ROD model of screw-rod. **c**, **d** The K-ROD model

**Fig. 4:**
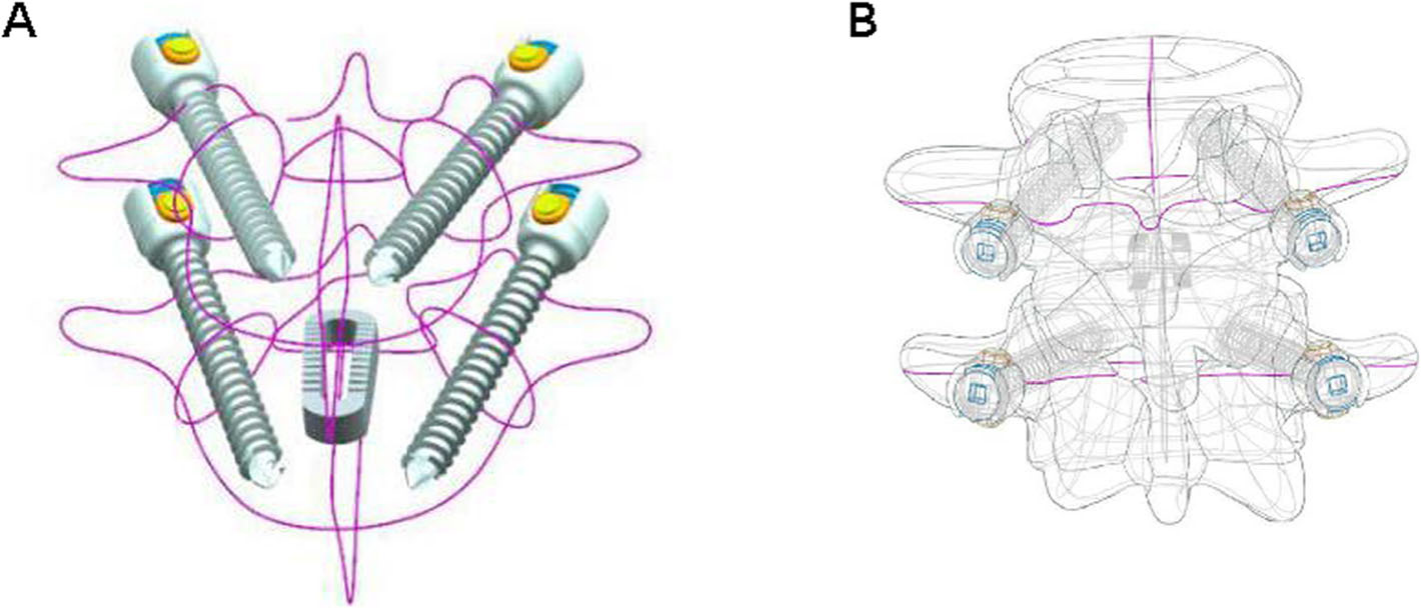
**a** The sketch of fusion cage and K-ROD assembly model. **b** The model of fusion cage and K-ROD assembly model

The nailrod model and the cage model were designed and assembled using the UG software. The cage was individually designed to make the upper and lower surfaces of the cage completely match the adjacent vertebral body surfaces. The Arbeitsgemeinschaftfür Osteosynthesefragen (AO) spinal internal fixation standard was used as the reference. The screws were implanted in the center of the pedicle and a 20° cohesion angle was used in the L4–L5 pedicle screws (Figs. 3 and 4).

**Fig. 5:**
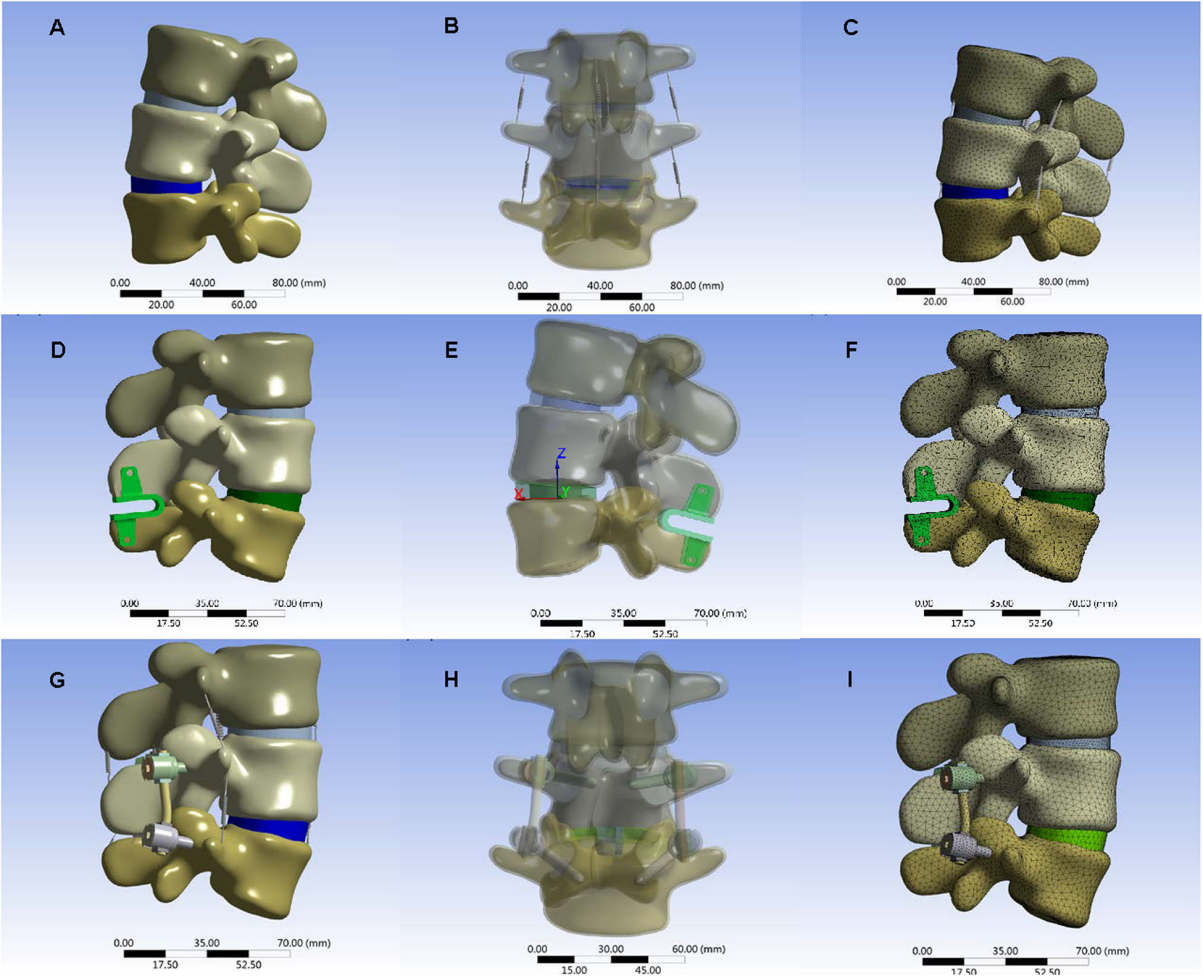
Assembly model **a**, **d**, **g** 3D stereogram view of normal lumbar, Coflex model, and PLIF model. **b**, **e**, **h** 3D perspective view of normal lumbar, Coflex model, and PLIF model. **c**, **f**, **i** 3D Grid diagram view of normal lumbar, Coflex model, and PLIF model

The normal lumbar spine, Coflex, and K-ROD assembly models were imported into ANSYS 17. Then, ANSYS analysis files were generated for each model (Fig. 5). Table 1 shows the material properties of the bones, intervertebral disc, implants, and various ligaments. In ANSYS, the simulation of ligaments was achieved by adding a spring unit (tension only). Table 2 shows the properties of the spring unit [10].

**Table 1:** Material properties of the tissues and implants

	Young’s modulus (MPa)	Poisson’s ratio	Element type	Reference
Cancellous bone	150	0.2	10-node tetrahedral solid element	[1, 2]
Cortical bone	18,000	0.3	10-node tetrahedral solid element	[1, 2]
Nucleus pulposus	2	0.45	10-node tetrahedral solid element	[1, 3]
Annulus fibrosus	8	0.49	10-node tetrahedral solid element	[1, 3]
Implant (Ti-6Al-4 V)	114,000	0.3	10-node tetrahedral solid element	[1, 2]
Bone-cage*	110,000	0.3	10-node tetrahedral solid element	[4, 5]

*Intervertebral fusion cage was used to supplement the K-ROD internal fixation system

**Table 2:** Ligament stiffness matrix in N/mm with the according ranges of the intervals

Ligament	ALL	PLL	ISL	SSL	LF	IT
L3–L4	39.5 ± 20.3	10.6 ± 8.5	18.1 ± 15.9	34.8 ± 11.7	34.5 ± 6.2	50.0
L4–L5	40.50 ± 14.3	25.8 ± 15.8	8.7 ± 6.5	18.0 ± 6.9	27.2 ± 12.2	50.0

*ALL* anterior longitudinal, *PLL* posterior longitudinal, *ISL* intraspinous, *SSL* supra-spinous, *LF* flavum, *IT* intertransverse

### Pre-compressive load

**Fig. 6:**
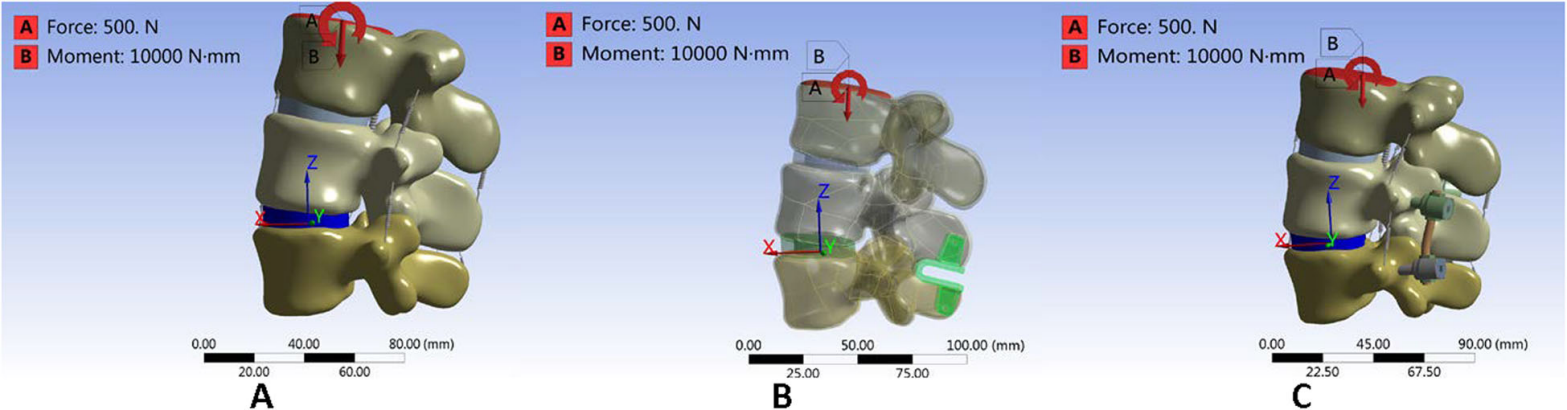
Load addition method. **a** Load addition method model in normal model. **b** Load addition method model in Coflex model. **c** Load addition method model in PLIF model

A 500-N pre-compressive load equal to two-thirds of the human body mass (50 kg, 500 N) [5] was applied on the upper surface of the L3 lumbar vertebra. An additional moment of 10 N*m (10,000 N*mm) was exerted in each direction as described in the literature [5–12] to simulate lumbar anteflexion, rear protraction, and rotation (Fig. 6).

### Simplified restriction

A six-degree-of-freedom rigidly fixed constraint was applied on the lower surface of the L5 lumbar vertebra according to the previous literature [11, 12] to verify that the lower surface of L5 vertebral would not produce displacement and rotation when moment was added.

### Bind

All ligaments were simplified into spring units as shown in Table 2. The BOND connection was used between the vertebral body and the intervertebral disc. The facet joint surface was subjected to frictional simulation and the friction coefficient was set as 0.2 [4–9, 11–13]. “No Separation” was used to connect the surfaces of Coflex with those of the spinous processes. The BOND connection was applied in all the K-ROD components, cage, and vertebral body connections.

### Meshing

To ensure the comparability of the models and prompt calculation, and also to avoid the calculation error caused by meshing, a 10-node tetrahedral mesh was used in mesh 7. A more refined mesh of 1.5 mm was used in L3/4 intervertebral disc and the inner implant. The octahedral mesh was used in the cancellous bone. The same meshing method was applied in the normal lumbar, Coflex, and PLIF implant models. The mesh of 3 mm was used in all the other parts. The meshing was performed using the ANSYS software (ANSYS WORKBENCH 15.0). The units used are shown in Table 1. No warnings and failures were reported. Table 3 shows the number of nodes and elements in the three models.

**Table 3:** Number of nodes and elements in the models

Model	Nodes	Elements
Normal	201654	136934
Coflex	211836	142718
K-ROD	345475	235081

## Verification method

### Verification of L4, L5 segment axial compression (displacement-load curve)

**Fig. 7:**
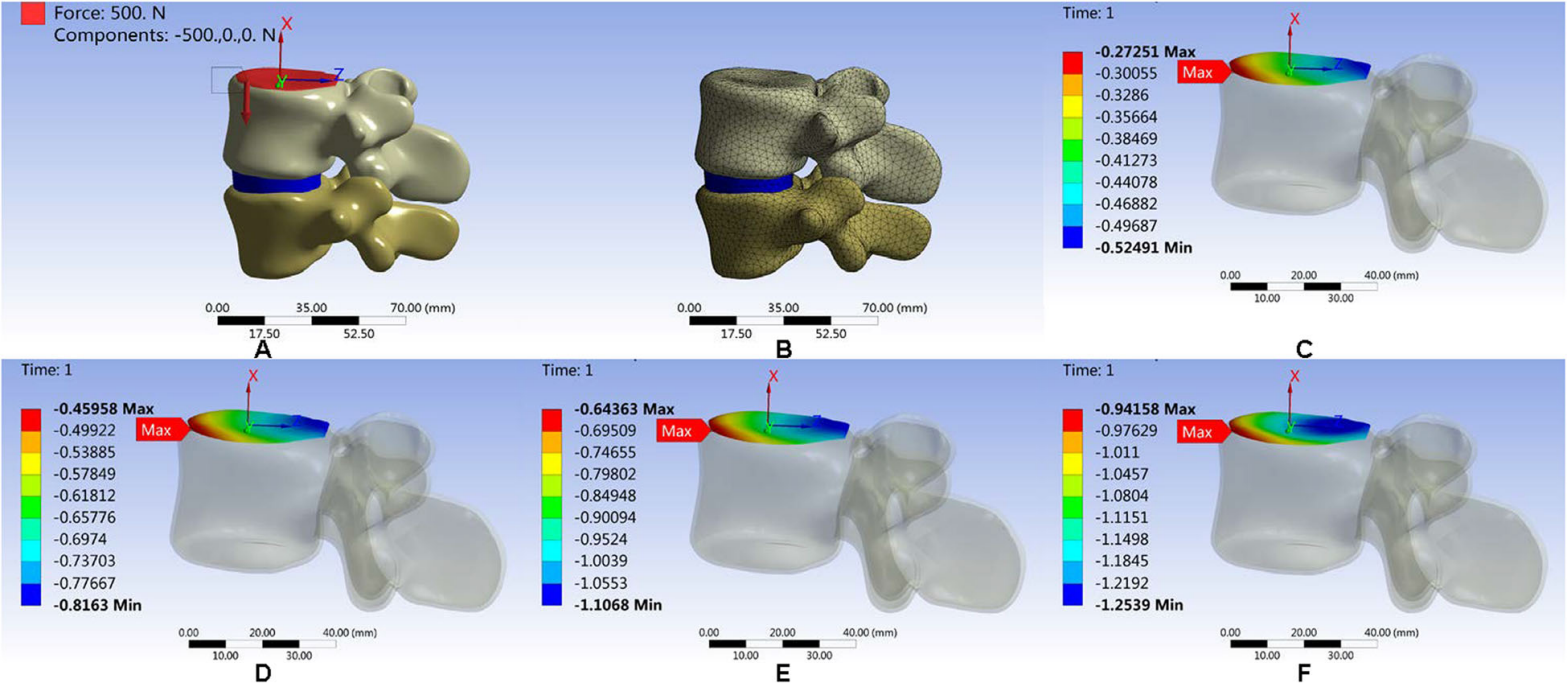
Axial displacement verification experiment. **a**, **b** Lumbar model. **c** Axial displacement of lumbar vertebral body model under 500 N pre-compressive load. **d** Axial displacement of lumbar vertebral body model under 1000 N pre-compressive load. **e** Axial displacement of lumbar vertebral body model under 1500 N pre-compressive load. **f** Axial displacement of lumbar vertebral body model under 2000 N pressure

After establishing the L4/L5 lumbar model, a pre-compressive load ranging from 500 to 2000 N was applied on the upper surface of L4. The axial displacement of the lumbar vertebral model was calculated and compared with the references [14–18], as shown in Fig. 7.

### Measurement of lumbar range of motion and torsional rigidity

The origin of the local coordinate system was defined as the center of endplate in the distal vertebral body. Perpendicular to the endplate in the sagittal position was the *X*-axis. Parallel to the endplate was the *Z*-axis (right) and the *Y*-axis (left).

With the distal vertebral body fixed, two nodes on the upper surface of the adjacent lumbar were selected. The coordinates of the two nodes before pre-compressive load application were (*X*1, *Y*1, *Z*1), (*X*2, *Y*2, *Z*2). The coordinates of the two nodes after pre-compressive load application were (*X*3, *Y*3, *Z*3), (*X*4, *Y*4, *Z*4). The lines connecting the two points before and after pre-compressive load application formed the angle *θ* (i.e., the maximum angular displacement) [11, 19–21]. The formula is presented as follows:

$$ \uptheta =\frac{180}{\pi}\kern0.5em \times \kern0.5em acrc\mathrm{os}\kern0.5em \frac{\left({X}_1-{X}_2\right)\times \left({X}_3-{X}_4\right)+\left({Y}_1-{Y}_2\right)\times \left({Y}_3={Y}_4\right)+\left({Z}_1-{Z}_2\right)\times \left({Z}_3-{Z}_4\right)}{\sqrt{{\left({X}_1-{X}_2\right)}^2+}{\left({Y}_1-{Y}_2\right)}^2+{\left({Z}_1-{Z}_2\right)}^2\times \sqrt{{\left({X}_3-{X}_4\right)}^2+{\left({Y}_3-{Y}_4\right)}^2+{\left({Z}_3-{Z}_4\right)}^2}} $$

Torsional rigidity was the moment required to produce a unit torsion of the lumbar vertebra and it was measured using the following formula: Torsional rigidity(*k*) = *M*/*θ*, (*M*: the exerted moment; *θ*:the range of motion).

### Verification of maximum von Mises equivalent stress on L3/L4 intervertebral disc

The uppermost L3 was not subject to any constraints. An evenly distributed 500-N pre-compressive load was exerted on the endplate of the L3 vertebral body; the motor moment was 10 Nm [8, 19, 20]. The maximum von Mises equivalent stress on the L3/L4 intervertebral disc was calculated by FE analysis and compared with the references.

### Evaluation of the sensitivity of the elements

**Fig. 8:**
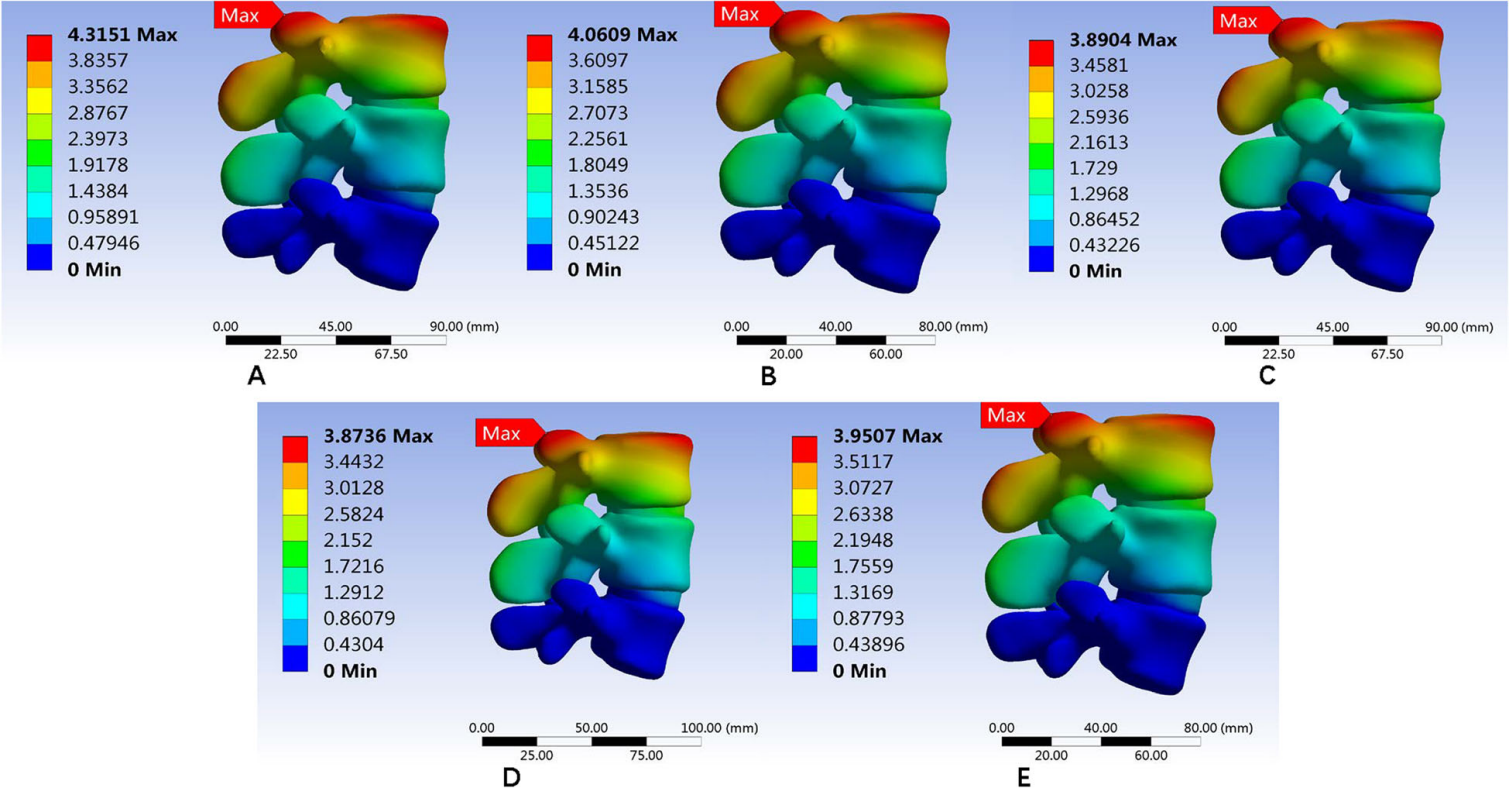
Evaluation of the sensitivity of the elements. **a** Low-medium. **b** Medium. **c** Medium-high. **d** Optimized. **e** High densities

The low-medium, medium, medium-high, optimized, and high densities of five representative meshes were performed to determine the number of elements (Fig. 8). An analysis was performed using the five densities, and the results were converged from the medium- to high-mesh densities. Table 4 shows the number of elements, number of nodes, computational time, max model displacement, and equivalent stress on the adjacent disc for each density.

**Table 4:** The sensitivity of the elements

Mesh	Nodes	Element	Approximate computational time (min)	Max model displacement (mm)	Adjacent disc von Mises (MPa)
Coarse	125970	84676	8	4.3151	1.734
Medium	181156	123871	15	4.0609	1.455
Fine	537548	375680	40	3.8904	1.8582
Very fine	942449	675635	180	3.8736	1.8219
Optimize	201654	136934	25	3.9507	1.8162

## Results

### Verification of experimental results

#### Verification of L4, L5 segment axial compression (displacement-load curve)

**Fig. 9:**
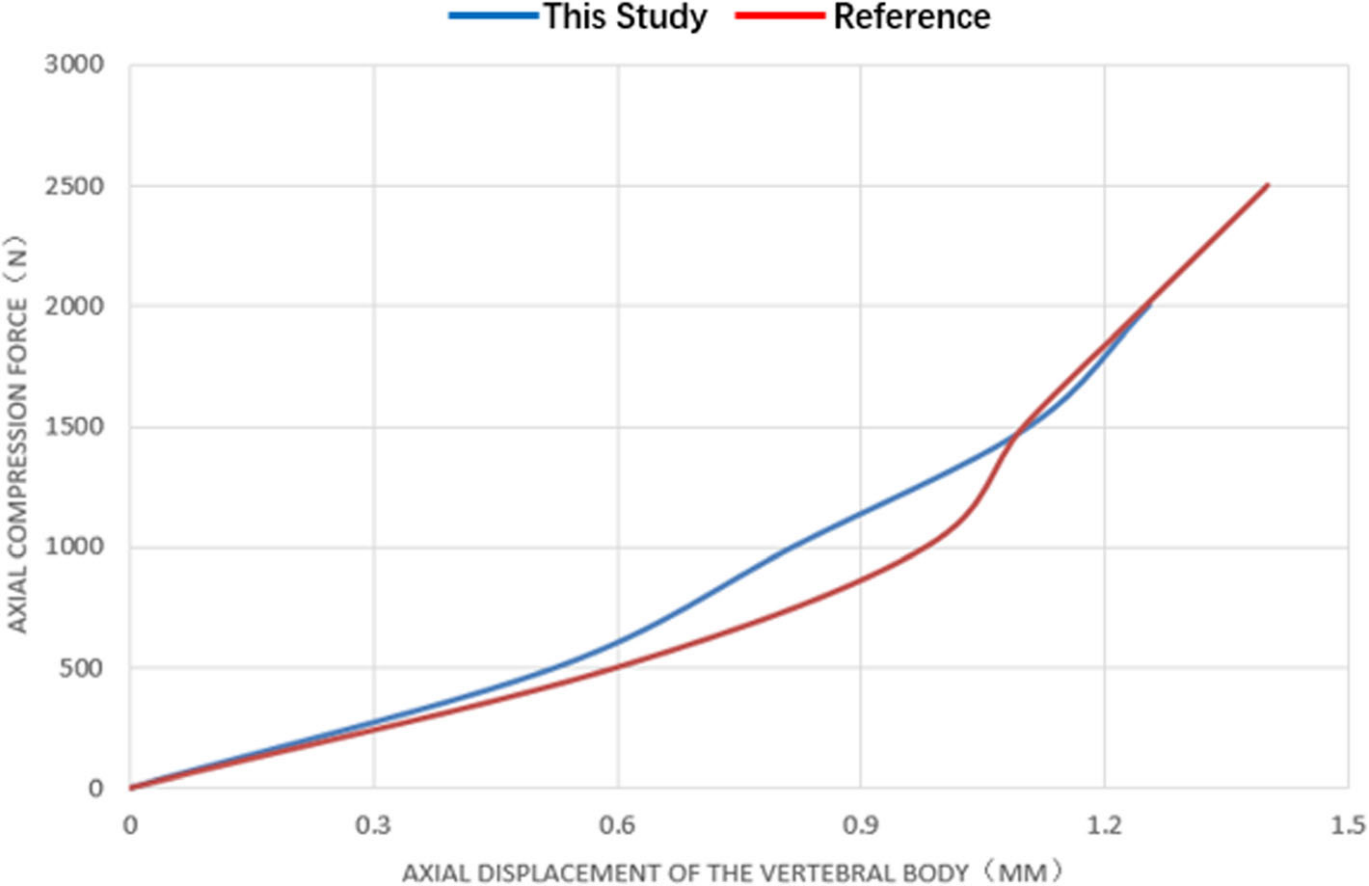
Displacement-load comparison curve between this study and reference

Compared with the results from the literature [14–18], ours was approximated to that of Virgin’s [18], and between those of Virgin’s and Markolf’s. The axial compression stiffness of our model was of an intermediate level among the results in the literature. The curve was obviously nonlinear, which was in accordance with the literature (Fig. 9).

### Measurement of lumbar ROM and torsional rigidity

The ROM and torsional rigidity of this study is shown in Table 5. The ROM and torsional rigidity of the models were in good accordance with those reported in the literature, as shown in Table 6.

**Table 5:** ROM and torsional rigidity in different motions of this study

Motion state	Vertebral	ROM (°)	Average ROM of L3 and L4 (°)	Rotational stiffness (N.m/°)	Average rotational stiffness of L3 and L4 (N.m/°)
Anteflexion 10(N*m)	L3	4.8739	3.4137	2.051745	3.585381
	L4	1.9535		5.119017	
Rear protraction 10(N*m)	L3	5.1141	3.88215	1.955378	2.864339
	L4	2.6502		3.7733	
Lateral bending 10(N*m)	L3	3.65885	3.037475	3.65885	3.450752
	L4	2.4161		2.4161	
Axial rotation 10(N*m)	L3	4.68015	3.50455	4.68015	3.312286
	L4	2.32895		2.32895

**Table 6:** Comparison of ROM and torsional rigidity (N.m/°)

	Load moment(N*m)	Anteflexion	Rear protraction	Lateral bending	Axial rotation	Average
JiaW Zhi	10	2.35	3.58	2.86	8.98	3.226875
DeS Zhang	10	1.62	3.03	2.5	4.45	
Vadapalli S	10	2.32	2.85	2.53	3.64	
YuF Huang	10	1.83	2.92	2.51	3.66	
This study	10	3.585381	2.864339	3.450752	3.3122857	3.329299

### Verification of maximum von Mises equivalent stress on L3/L4 intervertebral disc

The maximum von Mises equivalent stress on the L3–L4 intervertebral disc in this model was in accordance with those from the other literature (Table 7).

**Table 7:** von Mises stress in the intervertebral disc of L3–L4 comparison with other literatures (Mpa)

	Load moment(N*m)	Upright	Anteflexion	Rear protraction	Lateral bending	Axial rotation
ZhaoH Chen	10	–	1.951	3.037	1.916	1.831
Wang X	10	–	3.03	4.95	1.52	2.11
El Reich M	10	0.994	2.15	3.04	1.86	3.76
YuF Huang	10	0.978	2.92	2.329	1.917	2.405
This study	10	1.2671	1.8162	2.943	2.6322	2.1069

### Evaluation of the sensitivity of the elements

**Fig. 10:**
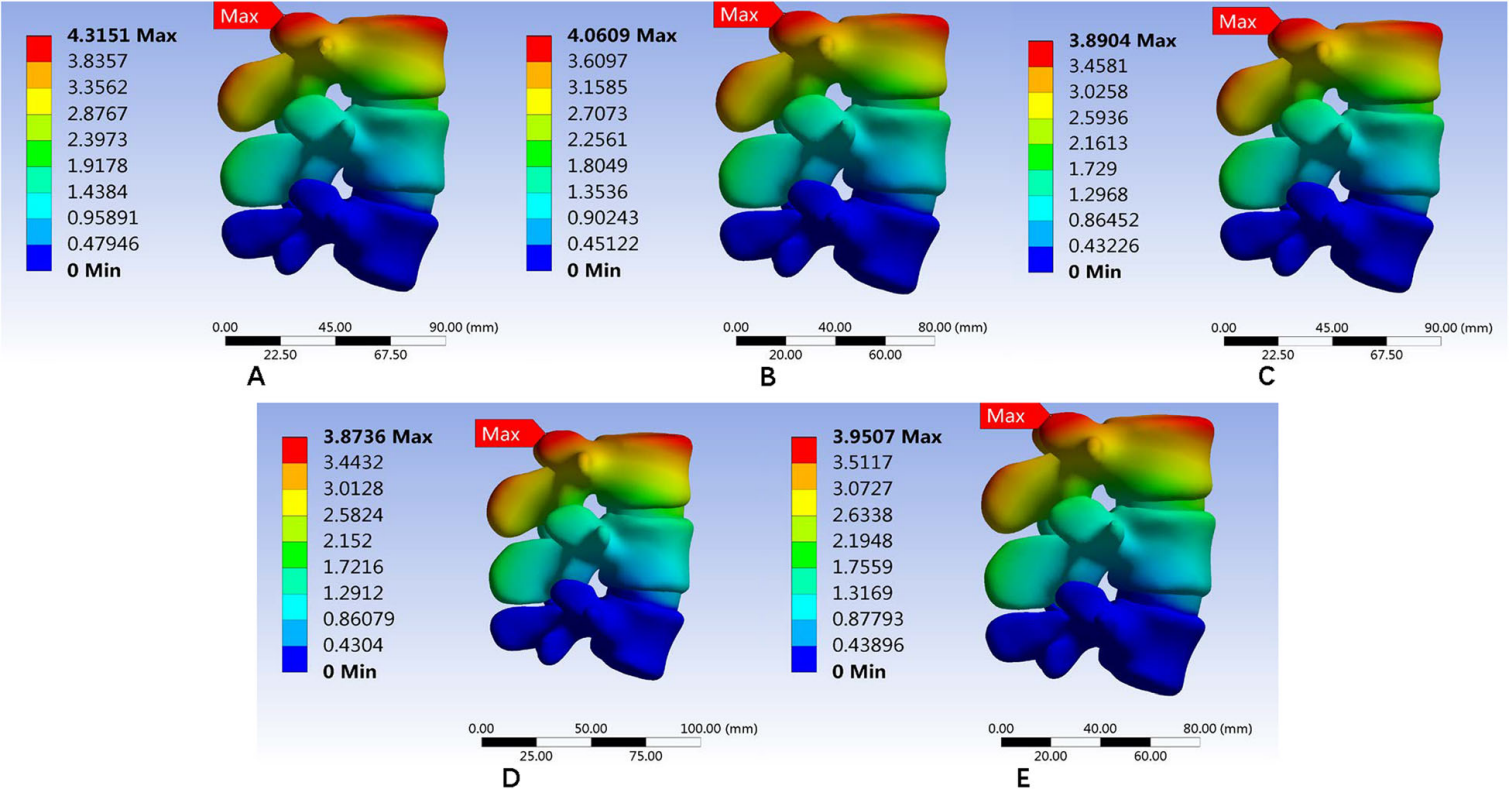
Classification according to the mesh densities to evaluate the sensitivity of the elements. **a** Coarse mesh. **b** Medium mesh. **c** Fine mesh. **d** Very fine mesh. **e** optimized mesh

For high and medium-high mesh, the convergence and accuracy were increased; however, they were not practiced on account of the increased computational time. Therefore, the optimized mesh with a high degree of convergence was selected for the analysis (Table 8, Fig. 10).

**Table 8:** Analysis of the state and influence on the interpretation by densities of the mesh

Mesh	Number of nodes	Number of elements	Approximate computational time (min)	Max model displacement (mm)	Stress on adjacent disc (Mpa)
Low-medium	125970	84676	8	4.3151	1.734
Medium	181156	123871	15	4.0609	1.455
Medium-high	537548	375680	40	3.8904	1.8582
High	942449	675635	180	3.8736	1.8219
Optimization	201654	136934	25	3.9507	1.8162

#### L3–L4 lumbar ROM and torsional rigidity

In the normal model, the lumbar ROM of the L3–L4 was higher than the others. The Coflex and PLIF models exhibited significant differences in anteflexion, rear protraction, and right lateral bending. The Coflex model had a significantly smaller L3–L4 axial rotation angle than the normal model, and the rotation in the upright position had the smallest ROM. The right axial rotation showed the largest ROM. However, the ROMs of upright, rear protraction, and left lateral bending showed no significant change compared with the PLIF model. In the PLIF model, the upright position had the smallest L3–L4 ROM and the biggest right axial rotation. The ROM of PLIF showed significant reductions, but the left lateral bending did not change significantly, compared with the normal and Coflex models (Table 9).

**Table 9:** Finite element analysis of L3–L4 lumbar ROM (°)

	Upright	Anteflexion	Rear protraction	Right lateral bending	Left lateral bending	Left axial rotation	Right axial rotation
Normal	1.8073	4.8739	5.1141	3.9043	3.4134	4.6831	4.6772
Coflex	1.1788	2.6823	2.1049	1.6505	3.1443	4.1651	4.6857
PLIF	1.0288	1.1214	2.1049	2.0873	3.1128	2.7848	3.287

In terms of the L3–L4 lumbar spine torsional rigidity, the PLIF model was significantly higher than the normal and Coflex models, and the Coflex model was higher than the normal. In the normal model, the rear protraction had the smallest stiffness and the left lateral bending had the largest stiffness. In the Coflex model, the stiffness of L3–L4 right axial rotation was the smallest and the right lateral bending rotation was the largest; compared with the normal, the stiffness of anteflexion, rear protraction, left lateral bending, and left axial rotation was significantly increased. The average L3–L4 lumbar torsional rigidity reached 3.708858 N*m/°, which was significantly larger than the normal model. In the PLIF model, compared with Coflex, L3–L4 lumbar torsional rigidity was significantly increased in all the positions except in rear protraction and right lateral bending. All the data are presented in Table 10.

**Table 10:** L3–L4 lumbar torsional rigidity (N*m/°)

	Anteflexion	Rear protraction	Right lateral bending	Left lateral bending	Left axial rotation	Right axial rotation	Average
Normal	2.051745	1.955378	2.561279	2.92963	2.135338	2.138031	2.295234
Coflex	3.728144	4.75082	6.05877	3.180358	2.400903	2.134153	3.708858
K-ROD	8.917425	4.75082	4.790878	3.212542	3.590922	3.042288	4.717479

#### Maximum von Mises equivalent stress on L3/L4 intervertebral disc

**Fig. 11:**
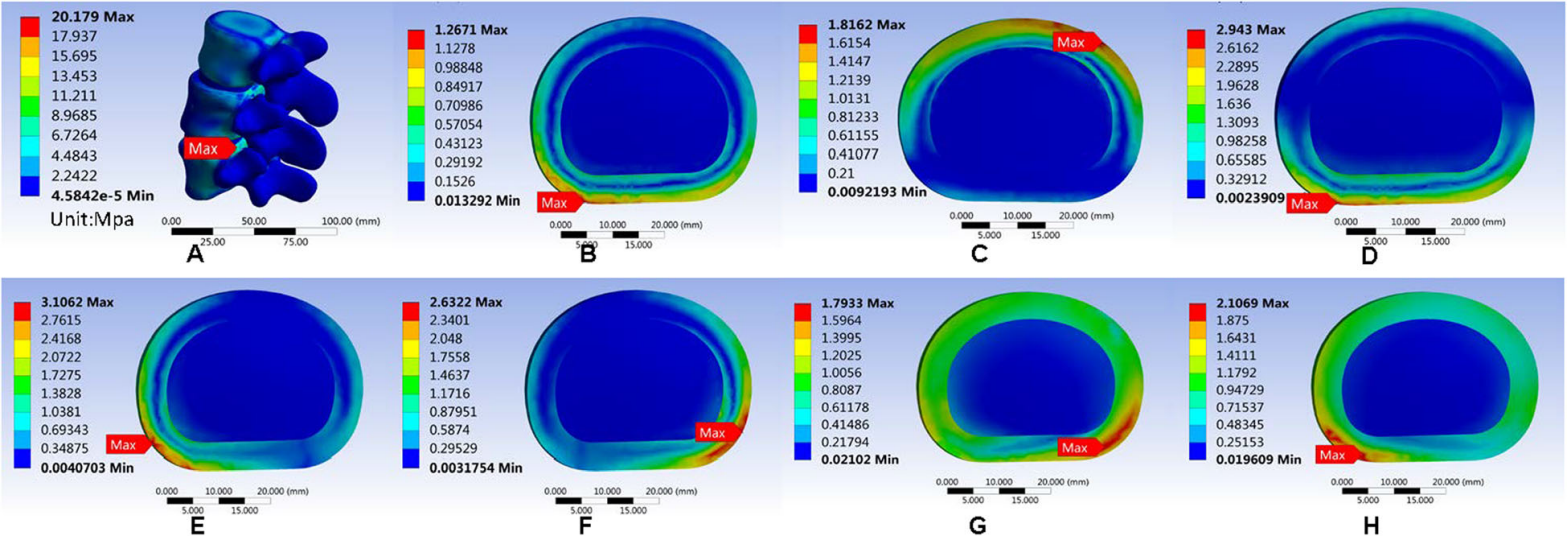
Stress distribution of the surgical segment (L3–L4) disc annulus in normal surgical model for various motions. **a** Maximum von Mises stress when standing on a lumbar spine model. **b** Maximum von Mises equivalent stress on L3/L4 intervertebral disc in standing posture. **c** Maximum von Mises equivalent stress on L3/L4 intervertebral disc in anteflexion. **d** Maximum von Mises equivalent stress on L3/L4 intervertebral disc in extension. **e** Maximum von Mises equivalent stress on L3/L4 intervertebral disc in right bending. **f** Maximum von Mises equivalent stress on L3/L4 intervertebral disc in left bending. **g** Maximum von Mises equivalent stress on L3/L4 intervertebral disc in left rotation. **h** Maximum von Mises equivalent stress on L3/L4 intervertebral disc in right rotation

**Fig. 12:**
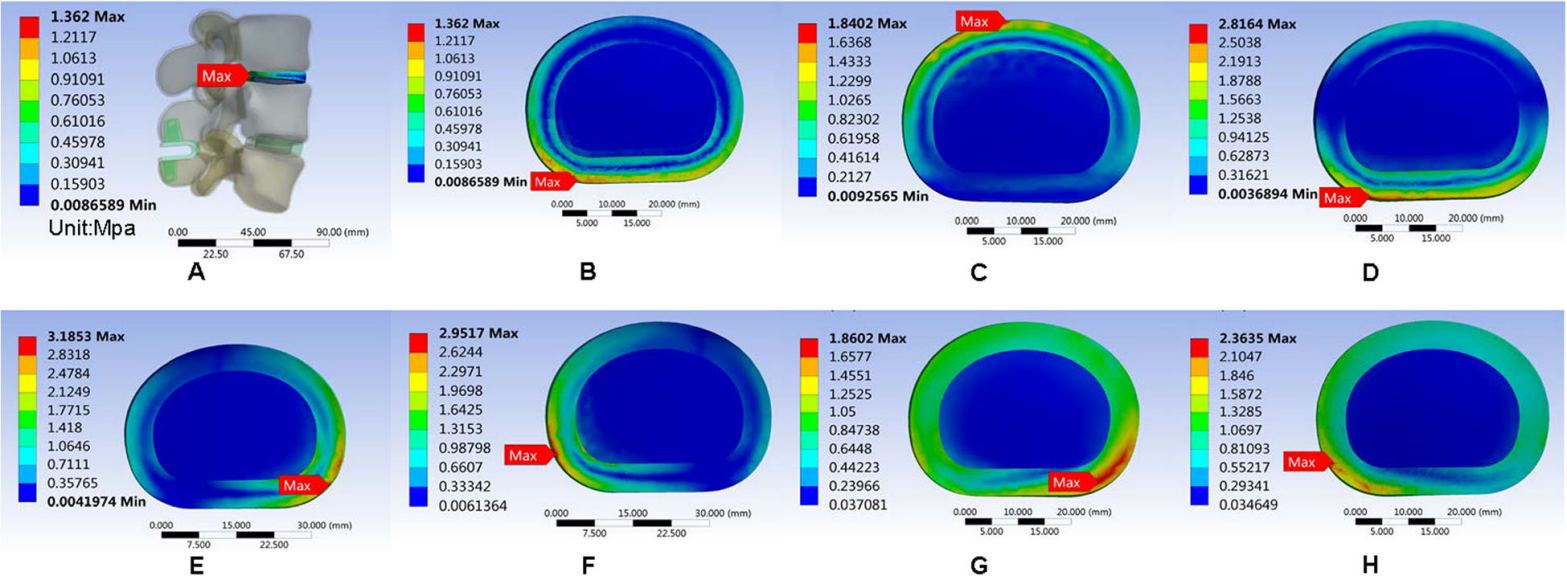
Stress distribution of the surgical segment (L3–L4) disc annulus in normal surgical model for various motions. **a**, **b** Maximum von Mises equivalent stress on L3/L4 intervertebral disc in standing posture. **c** Maximum von Mises equivalent stress on L3/L4 intervertebral disc in anteflexion. **d** Maximum von Mises equivalent stress on L3/L4 intervertebral disc in extension. **e** Maximum von Mises equivalent stress on L3/L4 intervertebral disc in right bending. **f** Maximum von Mises equivalent stress on L3/L4 intervertebral disc in left bending. **g** Maximum von Mises equivalent stress on L3/L4 intervertebral disc in left rotation. **h** Maximum von Mises equivalent stress on L3/L4 intervertebral disc in right rotation

**Fig. 13:**
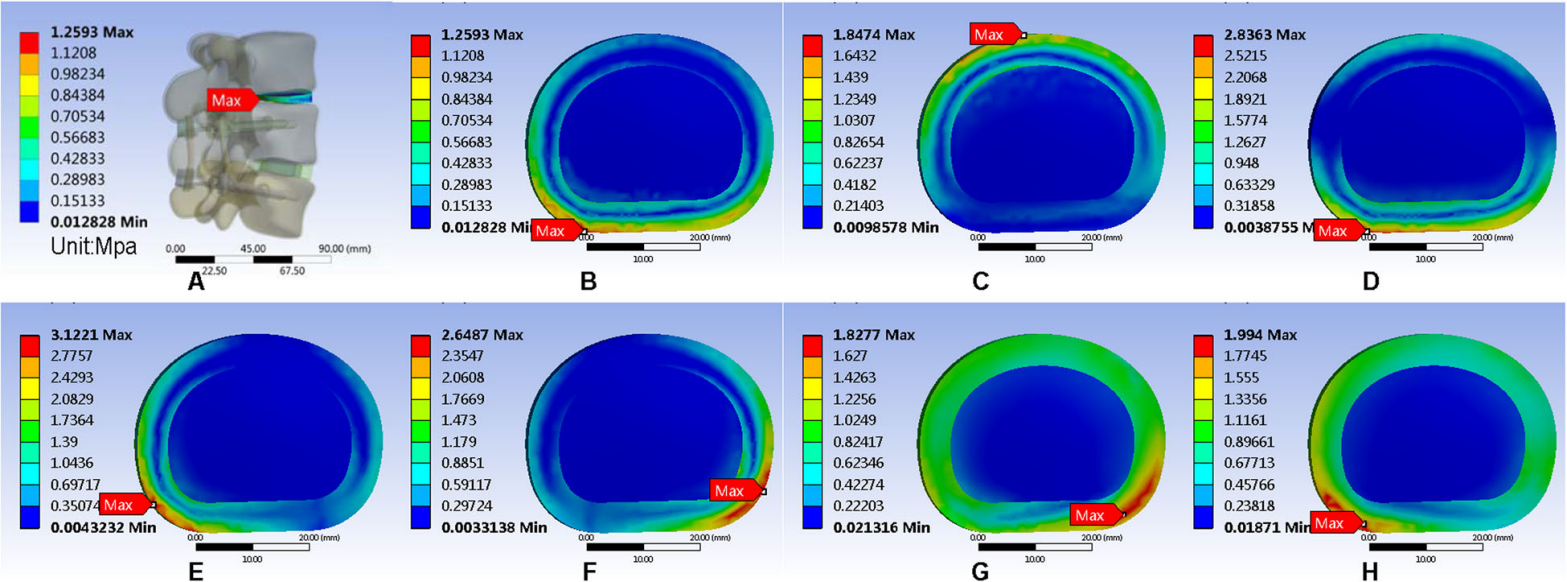
Stress distribution of the surgical segment (L3–L4) disc annulus in PLIF surgical model for various motions. **a**, **b** Maximum von Mises equivalent stress on L3/L4 intervertebral disc in standing posture. **c** Maximum von Mises equivalent stress on L3/L4 intervertebral disc in anteflexion. **d** Maximum von Mises equivalent stress on L3/L4 intervertebral disc in extension. **e** Maximum von Mises equivalent stress on L3/L4 intervertebral disc in right bending. **f** Maximum von Mises equivalent stress on L3/L4 intervertebral disc in left bending. **g** Maximum von Mises equivalent stress on L3/L4 intervertebral disc in left rotation. **h** Maximum von Mises equivalent stress on L3/L4 intervertebral disc in right rotation

The maximum von Mises equivalent stress on L3–L4 intervertebral disc showed no significant difference among the three models. In the normal model, the intervertebral disc had the largest maximum von Mises equivalent stress when in the right lateral bending position. The smallest stress appeared when in the upright position. The maximum von Mises equivalent stresses in all positions were smaller than the other two models (Fig. 11). In the Coflex group, the maximum von Mises equivalent stress of the intervertebral disc was the largest in right lateral bending and the smallest in the upright position (Fig. 12). The maximum von Mises equivalent stress of L3–L4 intervertebral disc in the PLIF model did not differ significantly from that of the Coflex model (Fig. 13). All the data are presented in Table 11.

**Table 11:** Maximum von Mises equivalent stress (Mpa) of L3/L4 intervertebral disc

	Upright	Anteflexion	Rear protraction	Right lateral bending	Left lateral bending	Left axial rotation	Right axial rotation
Normal	1.2671	1.8162	2.943	3.1062	2.6322	1.7933	2.1069
Coflex	1.362	1.8402	2.8164	3.1853	2.9517	1.8602	2.3635
K-ROD	1.2593	1.8474	2.8363	3.1221	2.6487	1.8277	1.994

#### Maximum von Mises equivalent stress of implant

**Fig. 14:**
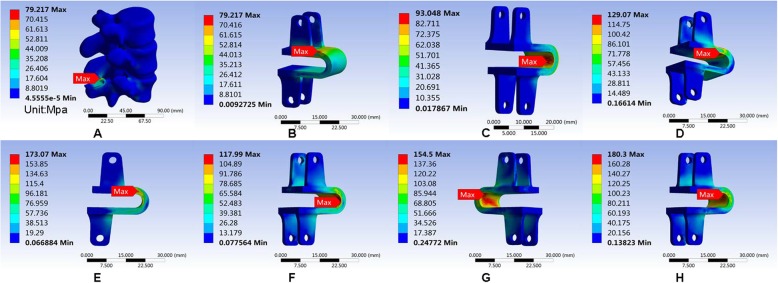
Stress distribution of the Coflex in Coflex surgical model for various motions. **b** Maximum von Mises equivalent stress on Coflex in standing posture. **a**, **c**: Maximum von Mises equivalent stress on Coflex in anteflexion. **d** Maximum von Mises equivalent stress on Coflex in extension. **e** Maximum von Mises equivalent stress on Coflex in right bending. **f** Maximum von Mises equivalent stress on Coflex in left bending. **g** Maximum von Mises equivalent stress on Coflex in left rotation. **h** Maximum von Mises equivalent stress on Coflex in right rotation

**Fig. 15:**
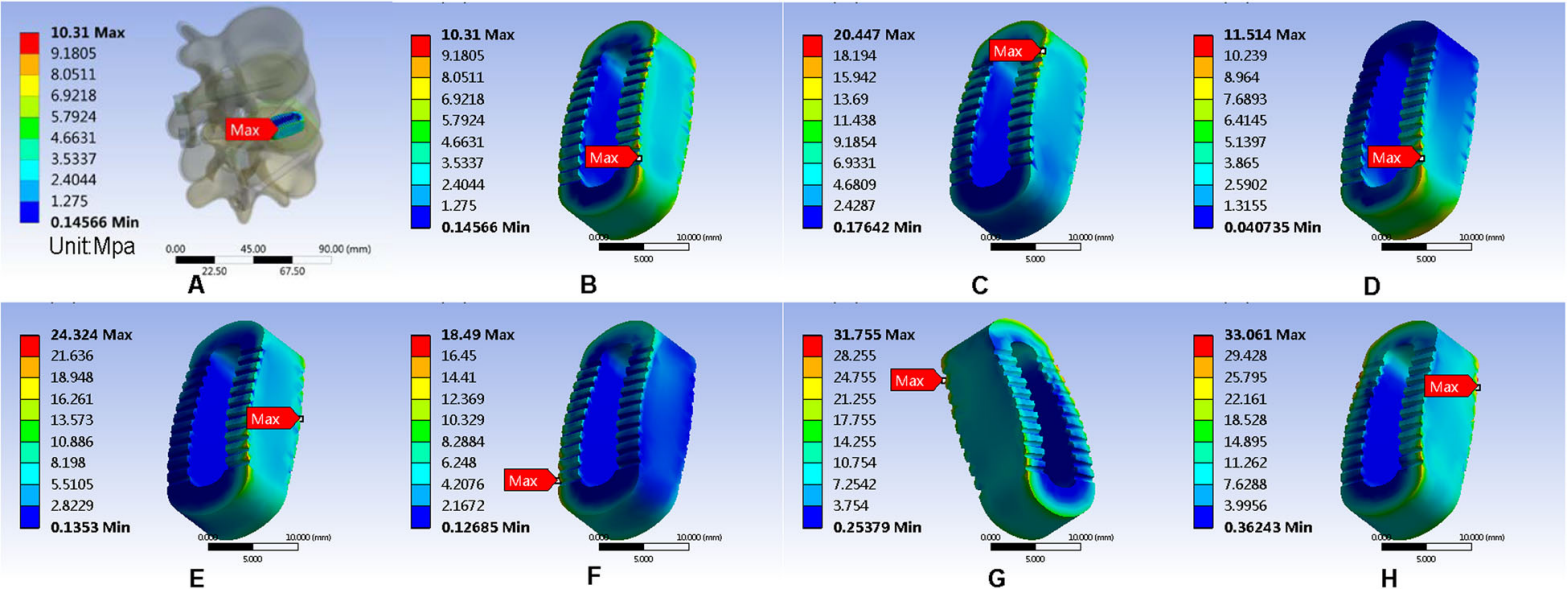
Stress distribution of the K-ROD (Cage) in PLIF surgical model for various motions. **a**, **b** Maximum von Mises equivalent stress on K-ROD (Cage) in standing posture. **c** Maximum von Mises equivalent stress on -ROD (Cage) in anteflexion. **d** Maximum von Mises equivalent stress on K-ROD (Cage) in extension. **e** Maximum von Mises equivalent stress on K-ROD (Cage) in right bending. **f** Maximum von Mises equivalent stress on K-ROD (Cage) in left bending. **g** Maximum von Mises equivalent stress on K-ROD (Cage). **h** Maximum von Mises equivalent stress on K-ROD (Cage) in right rotation

**Fig. 16:**
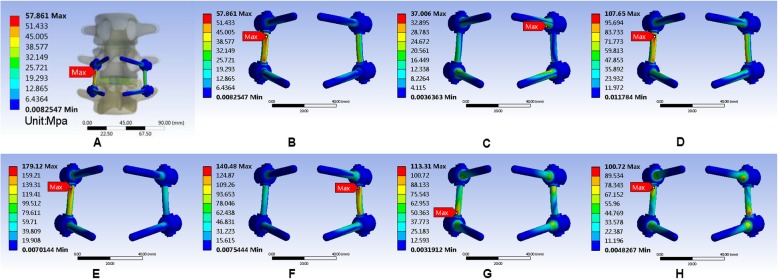
Stress distribution of the K-ROD (pedicle screw) in PLIF surgical model for various motions. **a**, **b** Maximum von Mises equivalent stress on K-ROD (pedicle screw) in standing posture. **c** Maximum von Mises equivalent stress on K-ROD (pedicle screw) in anteflexion. **d** Maximum von Mises equivalent stress on K-ROD (pedicle screw) in extension. **e** Maximum von Mises equivalent stress on K-ROD (pedicle screw) in right bending. **f** Maximum von Mises equivalent stress on K-ROD (pedicle screw) in left bending. **g** Maximum von Mises equivalent stress on K-ROD (pedicle screw) in left rotation. **h** Maximum von Mises equivalent stress on K-ROD (pedicle screw) in right rotation

In this study, we performed the finite element analysis of Coflex, cage, and nail rods in each model to measure the maximum von Mises equivalent stress of the implant. It showed that Coflex had the smallest maximum von Mises equivalent stress when in the upright position, and the smallest maximum von Mises equivalent stress appeared in the right axial rotation. The maximum von Mises equivalent stress of the cage was the smallest compared with the other two parts, and the smallest stress appeared in the upright position and the largest in right axial rotation. The smallest stress on the nail was in anteflexion, unlike the other two implants. The largest stress was at the right lateral bending (Figs. 14, 15, and 16). All data are presented in Table 12.

**Table 12:** Maximum von Mises equivalent stress (Mpa) of implants

	Upright	Anteflexion	Rear protraction	Right lateral bending	Left lateral bending	Left axial rotation	Right axial rotation
Coflex	79.217	93.048	129.07	173.07	117.99	154.5	180.3
Cage	10.31	20.447	11.514	24.324	18.49	31.755	33.061
Screw-rod	57.861	37.006	107.65	179.12	140.48	113.31	100.72

## Discussion

PLIF surgery, the “the golden standard” for severe LSS, can significantly relieve LSS symptoms (e.g., radical pain and palsy), but it can also accelerate the degeneration of the adjacent segment. Paul Park [21] reported a high incidence of ASD after PLIF surgery. According to two other studies, the incidence even reached 100% [22]. Shinya Okuda [22] reported a case of repeated ASD after PLIF in which the patient underwent four operations at different adjacent lumbar segments. Although the patient’s symptoms were resolved by the operation, the time between surgeries was increasingly shorter and the sacral slope decreased.

The “topping-off” surgical method—using either a hybrid stabilization device (HSD) or an inter-spinous process device (IPD)—is being widely used to treat ASD. Khoueir et al. [23] have classified posterior dynamic stabilization devices into three categories: (1) HSD with pedicle screw/rod instrument; (2) IPD such as Wallis and Coflex; (3) total facet replacement system. The device placed at the default adjacent segment after PLIF can effectively reduce the ROM and loading force on the adjacent segment. The systematic review by Po-Hsin Chou [24] showed that the fusion-alone group had a higher incidence of radiographic ASD (52.6%) and symptomatic ASD (11.6%) than revision surgery group (8.1%). Besides, the HSD and fusion groups had a higher incidence of radiographic ASD at the supra-adjacent segment (10.5% and 24.7%, respectively) than the IPD (1%). In a review of 91 cases, Lu et al. [25] evaluated and compared the incidence of ASD in the PLIF and the “topping-off” (an IPD was put at the adjacent segment proximal to the PLIF instrument) groups, showing that radiographic ASD occurred in 20 cases (48%) in the PLIF group and 3 (6%) in the “topping-off” group. The PLIF group had 9 symptomatic cases while only 3 were found in the “topping-off” group. He concluded that the “topping-off” device reduced the risk of ASD. Both PLIF and Wallis relieved the LSS symptoms [25]. In addition, Wallis significantly reduced the adjacent segment degeneration.

Coflex is an inter-spinous fixation device for the “topping-off” technology. It is an elastic U-shaped structure implanted after possessing the inter-spinous and supra-spinous ligaments. Qu SD [26] believed that Coflex relieved the nerve root compression by propping up the spinous process, maintaining the lordosis of the implanted segment, and reducing the ligamentum flavum invasion of the spinal canal. The distraction force also enlarged the intervertebral foramen and reduced the load on the intervertebral disc and the facet joint in flexion. The meta-analysis by Li et al. [27] showed that Coflex was more effective than PLIF in terms of decompression, the Oswestry Disability Index (ODI), length of hospital stay (LOS), and blood loss. Yuan et al. [28] held that Coflex surgery had significantly less blood loss, shorter hospital stay, and operative time than PLIF (*p* < 0.001), and it also had a lower reoperation rate for ASD than PLIF, yet with no statistical difference (11.1% vs. 4.8%, *p* = 0.277). He also thought Coflex was not suitable for the patients with lumbar disc herniation.

To investigate the mechanism of Coflex in reducing ASD incidence, mechanical analysis of the supra-lumbar segment is needed. Finite element analysis can simulate real physical systems (geometry and load cases) and perform the measurement using a mathematical approximation program. With simple and mutual elements, a finite unknown quantity can approximate a real system of infinitely unknown quantity.

In this study, no significant difference of intervertebral disc load force on the adjacent segment was found between Coflex and PLIF under a 500-N pre-compressive load. The increased adjacent disc pressure of Coflex ranged from − 5 to 10%, while the pressure of PLIF ranged from − 10 to 2%, indicating that the higher load force on the adjacent disc was not a primary risk factor of ASD.

Coflex significantly reduced the anteflexion and rear protraction ROM of the adjacent segment, and the lateral bending was also decreased. No change was found in the angle of axial rotation. By comparison, PLIF reduced the ROM in all motions. The decreased ROM promoted lumbar stability in both Coflex and PLIF models. Two factors might contribute to the difference in Coflex activity between our results and those from the literature: first, ours was an idealized model and the micro-motion of the Coflex facet was smaller than the actual one; second, the intervertebral facet joint in our model was intact, unlike those in the literature, which were partially resected.

The increased torsional rigidity can impose a heavier burden on the adjacent segment. PLIF increases the torsional rigidity, which means the patient may have a higher load burden on the adjacent disc and facet joint in rotational activity. The higher load burden would then lead to degeneration of the adjacent intervertebral segments and the nucleus pulposus would be prone to protrusion and nerve root compression. As a result, the patients receiving PLIF would have a higher incidence of ADS and suffer from nerve root compression. Hence, higher torsional rigidity could be a primary risk factor of ASD.

Rigorous conclusions cannot be made based on the results of this study because of the small sample size and the limited number of working conditions for each finite element model. A larger sample size and more working conditions should be included in the further study. The maximum von Mises equivalent stress on the intervertebral disc needs to be statistically analyzed and compared. A comprehensive comparison of the effects of Coflex and PLIF on adjacent segments is also necessary in the future study.

## Conclusion

Both Coflex and PLIF reduced lumbar ROM and therefore provided stability at the surgical and adjacent segments. Under the same pressure, both devices had almost the same maximum von Mises stress as the normal model on the adjacent intervertebral disc. But it is worthy to notice that the torsional rigidity of both PLIF and Coflex models was higher than that of the normal model, and PLIF produced an even higher burden on the adjacent segment than Coflex. It indicates that the lumbar vertebra of patient treated with PLIF undertakes a larger load to reach ROM of Coflex. Therefore, we presumed that ADS was related to higher torsional rigidity.

## Data Availability

The datasets used and/or analyzed during the current study are available from the corresponding author on reasonable request.

## Abbreviations

AO: Arbeitsgemeinschaftfür Osteosynthesefragen
ASD: Adjacent segment disease
DVT: Deep vein thrombosis
FE: Finite element
HSD: Hybrid stabilization device
IPD: Inter-spinous process device
LOS: Length of hospital stay
LSS: Lumbar spinal stenosis
ODI: Oswestry Disability Index
PLIF: Posterior lumbar interbody fusion

## References

[1] Ishimoto Y, Yoshimura N, Muraki S (2012). Prevalence of symptomatic lumbar spinal stenosis and its association with physical performance in a population-based cohort in Japan: the Wakayama Spine Study. Osteoarthritis Cartilage.

[2] Deyo RA, Mirza SK, Martin BI (2010). Trends, major medical complications, and charges associated with surgery for lumbar spinal stenosis in older adults. JAMA.

[3] Zaina F, Tomkins-Lane C, Carragee E (2016). Surgical versus non-surgical treatment for lumbar spinal stenosis. Cochrane Database Syst Rev.

[4] Lee N, Shin DA, Kim KN (2016). Paradoxical radiographic changes of Coflex Interspinous device with minimum 2-year follow-up in lumbar spinal stenosis. World Neurosurg.

[5] Ming CZ, Song MH, Jie Z (2010). Three—dimensional finite element analyses of unilateral pedicles crews fixation in lumbar spine. Chin J Spine Spinal Cord.

[6] Yang M, Zeng C, Li L (2018). Establishment of 3D-finite element model for analysis of biomechanical stability of extraforaminal or transforaminal lumbar interbody fusion. J Tongji Univ Med Sci.

[7] Yang M, Zeng C, Li L (2018). Biomechanical analyses of extraforaminal lumbar interbody fusion [J]. J Tongji Univ Med Sci.

[8] Jia-zhi Y, Wu Z-h, Wang X-s (2009). Finite element analysis on stress change of lumbar spine. Natl Med J China.

[9] Hao J, Piao Z, Li J (2012). Establishment of a normal human lumbar three-dimensional finite element model based on CT image and reverse engineering methods. J Clin Rehabilitative Tissue Eng Res.

[10] Putzer M, Auer S, Malpica W (2016). A numerical study to determine the effect of ligament stiffness on kinematics of the lumbar spine during flexion. BMC Musculoskelet Disord.

[11] Shin JK, Lim BY, Goh TS (2018). Effect of the screw type (S2-alar-iliac and iliac), screw length, and screw head angle on the risk of screw and adjacent bone failures after a spinopelvic fixation technique: a finite element analysis. PLoS One.

[12] Zhao Y, Li J, Wang D, Liu Y, Tan J, Zhang S (2012). Comparison of stability of two kinds of sacro-iliac screws in the fixation of bilateral sacral fractures in a finite element model. Injury.

[13] Driscoll M, Aubin C-E, Moreau A, Parent S (2011). Biomechanical comparison of fusionless growth modulation corrective techniques in pediatric scoliosis. Med Biol Eng Comput.

[14] Schultz AB, Warwich DN, Berkson MH (1979). Mechanical properties of human lumbar spine motion segments-Part I: responses in flexion,extension,lateral bending,and torsion. J Biomech Eng.

[15] Andersson GB, Schultz AB (1979). Effects of fluid injection on mechanical properties of intervertebral discs. J Biomech.

[16] Tencer AF, Ahmed AM, Burke DL (1982). Some static mechanical properties of the lumbar intervertebral joint, intact and injured. J Biomech Eng.

[17] Wilke HJ, Neef P, Caimi M (1999). New in vivo measurements of pressures in the intervertebral disc in daily life. Spine.

[18] Virgin WJ (1951). Experimental investigations into the physical properties of the intervertebral disc. J Bone Joint Surg Br.

[19] Huang Y (2015). Establishment of normal lumbosacral vertebral three-dimensional finite element. Orthop Biomech Mater Clin Study.

[20] Vadapalli S, Sairyo K, Goel VK (2006). Biomechanical rationale for using polyetheretherketone (PEEK) spacers for lumbar interbody fusion:a finite element study. Spine.

[21] Park P, Garton HJ, Gala VC (2004). Adjacent segment disease after lumbar or lumbosacral fusion: review of the literature. Spine.

[22] Okuda S, Oda T, Yamasaki R (2014). Repeated adjacent-segment degeneration after posterior lumbar interbody fusion. J Neurosurg Spine.

[23] Khoueir P, Kim KA, Wang MY (2007). Classification of posterior dynamic stabilization devices. Neurosurgical Focus.

[24] Po-Hsin C, Hsi-Hsien L, An HS (2017). Could the topping-off technique be the preventive strategy against adjacent segment disease after pedicle screw-based fusion in lumbar degenerative diseases? A systematic review. Biomed Res Int.

[25] Lu K, Liliang PC, Wang HK (2015). Reduction in adjacent-segment degeneration after multilevel posterior lumbar interbody fusion with proximal DIAM implantation. J Neurosurg Spine.

[26] Qu SD, Hai Y, Su QJ, Qu SP (2015). Finite element analysis of the refined interspinous dynamic system based on Coflex. Zhongguo Zuzhi Gongcheng Yanjiu.

[27] Li AM, Li X, Yang Z (2017). Decompression and coflex interlaminar stabilisation compared with conventional surgical procedures for lumbar spinal stenosis: a systematic review and meta-analysis. Int J Surg.

[28] Yuan W, Su QJ, Liu T (2017). Evaluation of Coflex interspinous stabilization following decompression compared with decompression and posterior lumbar interbody fusion for the treatment of lumbar degenerative disease: a minimum 5-year follow-up study. J Clin Neurosci.

